# Molecular Characterization of Shiga Toxin-Producing *Escherichia coli* Isolated from Ruminant and Donkey Raw Milk Samples and Traditional Dairy Products in Iran

**DOI:** 10.1100/2012/231342

**Published:** 2012-07-31

**Authors:** Hassan Momtaz, Rahil Farzan, Ebrahim Rahimi, Farhad Safarpoor Dehkordi, Negar Souod

**Affiliations:** ^1^Department of Microbiology, College of Veterinary Medicine, Islamic Azad University, Shahrekord Branch, Shahrekord 166, Iran; ^2^Young Researchers Club, Islamic Azad University, Shahrekord Branch, Shahrekord 166, Iran; ^3^Department of Food Hygiene, College of Veterinary Medicine, Islamic Azad University, Shahrekord Branch, Shahrekord 166, Iran; ^4^Young Researchers Club, Islamic Azad University, Jahrom Branch, Jahrom 74135-355, Iran

## Abstract

The aims of the current study were to detect the virulence factors and antibiotic resistance of Shiga toxin-producing *E. coli*, in animal milk and dairy products in Iran. After *E. coli* dentification with culture method, PCR assay were developed for detection of pathogenic genes, serotypes and antibiotic resistance genes of *E. coli*. Results showed that out of 719 samples, 102 (14.18%) were confirmed to be positive for *E. coli* and out of 102 positive samples, 17.64% were O26 and 13.72% were O157 and 1.96% were O91 and 1.96% were O145 serotypes. Totally, the prevalence of *stx*1 and *papA* genes were the highest while the prevalence of *sfaS* and *fyuA* were the lowest in the positive samples. PCR results showed that *tetA, tetB* were the highest (64.70%) and *aac*(3)-*IV* were the lowest (27.45%) antibiotic resistant genes in *E. coli* positive samples. Our study indicated that the isolated *E. coli* trains in these regions had a highest antibiotic resistance to tetracycline (58.82%) and the lowest to nitrofurantoin (3.92%). *tetA* gene and *E. coli* O157 serotype had highest and *aac*(3)-*IV* gene, and *E. coli* O145 serotype had a lowest frequency rates of antibiotics resistance genes, in the region.

## 1. Introduction

*Escherichia coli* (*E. coli*) is a gram-negative, rod-shaped, flagellated, nonsporulating, and facultative anaerobic bacterium which belongs to Enterobacteriaceae Family. This bacterium classified into several categories based on its virulence factors such as enterotoxigenic *E. coli* (ETEC), attaching and effacing *E. coli* (AEEC), enteropathogenic *E. coli* (EPEC), enterohemorrhagic *E. coli* (EHEC), and Shiga toxin-producing *E. coli* (STEC or VTEC) [1–5]. On the other hand, pathogenic *E. coli* are classified into groups of strains that cause a common disease using common and remarkable assortments of virulence factors that called pathotypes [6]. The Shiga toxin-producing *E. coli* (STEC) are the causative agents of severe clinical syndromes in humans like HUS. Four of the most important virulence factors of STEC are the two phage-encoded cytotoxins, called Shiga toxin 1 (*stx1*) and Shiga toxin 2 (*stx2*), the protein intimin (*eae*), and the plasmid-encoded enterohaemolysin or enterohaemorrhagic *E. coli* haemolysin (*ehly*) [7]. Gastrointestinal diseases, food poisoning, and even death in some cases make this microorganism an insidious threat to human health and food safety [4, 8]. Trough outbreaks in the European Union [9] and several reports of septicemia in human and animals [10, 11] up to recent studies in England [12], all of them have doubled the importance of addressing the issue.

Studies showed that raw animal milk samples and different dairy products are considered the primary reservoir of STEC [4, 13, 14]. Based on our knowledge, in the majority of cases STEC outbreaks were associated with consumption of raw seafood products, traditional dairy products, unpasteurized milk, contaminated food with pollution sources such as feces, contaminated water, contaminated equipments, infected water, and even infected chief, fast-food, contaminated plants' food, and finally raw or even undercooked foods ([15–18]. There are many *E. coli* serogroups such as O157, O26, O103, O111, O145, O45, O91, O113, O121 and O128 which produce Shiga toxins [16, 17, 19–21].

Reports showed that among the non-O157 STEC, O26 has been the most common serogroup [22]. Other studies indicated that O157, O26, and O111 serogroups have been responsible for many outbreaks of HC and HUS cases [1]. Another researches recommended that *E. coli* O157 is the most commonly implicated in outbreaks while O111, O26, O103, and O145 serotypes have also been associated with HC and HUS [2].

There are different cultural methods for enrichment and detection of STEC [23]; however none of them guarantee the speed, accuracy, sensitivity, specificity, and safety. On the other hand, many studies were advertising the safety, accuracy, sensitivity, and specificity of molecular methods like polymerase chain reaction (PCR) for detection of STEC [4, 18]. To the author's knowledge, in the majority of cases, if the treatment was not sufficient, diseases caused by *E. coli*, in addition to weakening the body immunity and susceptibility to other diseases, it can lead to death. Treatment of diseases caused by this bacterium often requires antimicrobial therapy; however antibiotic-resistant strains of bacteria cause more severe diseases for longer periods of time than their antibiotic-susceptible counterparts. Several studies showed that antibiotic resistance in *E. coli* is increasing in these days [24]. Therefore, identification of resistance genes of bacteria seems to be so essential in reduction of treatment costs.

Therefore, the purpose of the current survey was to study the prevalence of virulence factors and antibiotic resistance properties of Shiga toxin-producing *E. coli* isolate from bovine, ovine, caprine, buffalo, camel, and donkey raw milk samples and traditional dairy products in Iran.

## 2. Materials and Methods

### 2.1. Sampling and Detecting **E. coli**

Overall 424 milk samples were collected: bovine (*n* = 110), caprine (*n* = 85), ovine (*n* = 98), buffalo (*n* = 52), camel (*n* = 44), and donkey (*n* = 35) raw milk samples were collected from farm bulk tanks and milk collection centers from several geographic regions of Iran, from March 2010 to March 2011. Bovine and buffalo milk samples were collected throughout this time period. Because the lactating periods of ewes, donkeys, and goats in Iran is seasonal (from March through May and September to November of the subsequent year), caprine, donkey, and ovine milk samples were only available through these months within the forementioned time frame. At each site, sampling of milk was performed according to the International Dairy Federation Guidelines [34]. Samples (100 mL, in sterile glass containers) were transported to the laboratory at ca. 4°C within a maximum of 6–12 h after sampling.

For dairy products, 106 samples of soft cheese, 99 soft butter, and 90 soft ice-cream made up of raw milk were purchased from Shiraz, Isfahan, and Shahrekord—three big cities of Iran supermarkets. All of these dairy products were made traditionally and after collection were kept under refrigeration in plastic bags; information about dates of production and of assigned shelf-lives was not presented. Dairy product samples were collected over a period of six months between May and November 2010 and were analyzed on the day of acquisition. Samples were transported under refrigeration (4–6°C) in thermal boxes containing ice packs and were tested immediately after collection. A 25 g portion of each sample was blended with 225 mL of nutrient broth (Merck, Germany) for 2 min at normal speed, using a Stomacher lab blender and incubated at 37°C for 24 h. A 1 mL sample of the nutrient broth culture was mixed with 9 mL of MacConkey broth (Merck, Germany) and further incubated at 37°C for 24 h. One loop of each tube was streaked on MacConkey agar (Merck, Germany). Four colonies from each plate with typical *E. coli* morphology were selected and examined by biochemical tests, including hydrogen sulphide, citrate, urease, and indol.

### 2.2. DNA Isolation

Bacterial strains were overnight grown in trypticase soy agar (TSA—Merck, German) at 37°C. One colony was suspended in 100 *μ*L of sterile distilled water. After boiling the suspension for 13 min, this was followed by freezing and subsequently centrifuged at 14,000 rpm for 15 min to pellet the cell debris [35]. The supernatant was used as a template for amplification reaction.

### 2.3. Polymerase Chain Reaction

PCR assays for detection of STEC serotypes, virulence factors, and antibiotic resistance genes in *E. coli* isolated from animal milk samples and traditional dairy products were performed in 3 states as follows.

The PCR assays, specific primer sequences and the predicted size of the amplified products for the different pathogenic gene coding regions including, *cnf1, cnf2, stx1, stx2, eaeA, cdtB, papA, sfaS, fyuA, iutA, traT,* and *hlyA* were employed as previously described [36–40]. Strains of *E. coli* O157:K88ac:H19, CAPM 5933 and *E. coli* O159:H20, CAPM 6006 were used as positive controls.

Another PCR was performed for detection of *E. coli* serotypes by application of specific primers shown in Table 1. The PCR reaction was performed in a total volume of 50 *μ*L containing 2.5 *μ*L of DNA template, 1.5 mM MgCl_2_, 200 *μ*M dNTP (Fermentas), 0.5 *μ*M of each primers (F & R), 1.25 U Taq DNA polymerase (Fermentas), and 5 *μ*L PCR buffer 10X. Reactions were initiated at 1 cycle 95°C for 3 min, followed by 30 cycles of 95°C for 20 s, 58°C for 40 s, 72°C for 30 s, and a final elongation step at 72°C for 8 min, in a DNA thermal cycler for detection of O157, O145, O103, O26, and O111 serotypes and 5 *μ*L PCR buffer 10X, 2 mM Mgcl_2, _150 *μ*M dNTP, 0.75 *μ*M of each primers F & R, 1.5 U Taq DNA polymerase, and finally 3 *μ*L DNA template with initiated at 1 cycle 94°C for 6 min, followed by 34 cycles of 95°C for 50 s, 58°C for 70 s, 72°C for 55 s, and a final elongation step at 72°C for 5 min, for detection of O91, O128, O121, O113, and O45 serotypes of *E. coli*.

The presence of genes associated with resistance to streptomycin, tetracycline, trimethoprim, fluoroquinolone, gentamicin, sulfonamide, cephalothin, ampicillin, and chloramphenicol was determined by PCR, and a set of primers was used for each gene showed in Table 2. PCR reactions were performed in a total volume of 25 *μ*L, including 2 mM MgCl_2_, 50 mM KCl, 10 mM Tris-HCl (pH 9.0), 0.1% Triton X-100, 150 *μ*M dNTPs each, 0.4 *μ*M primers, 1 U of Taq DNA polymerase, and 4 *μ*L (40–260 ng/*μ*L) of DNA. Amplification reactions were carried out using a DNA thermocycler as follows: three min at 95°C, 35 cycles each consisting of 1 min at 94°C, 90 s at ~55°C (show in Table 2), 1 min at 72°C, and a final extension step of 8 min at 72°C.

Finally, a DNA thermocycler (Eppendorf Mastercycler, Eppendorf-Nethel-Hinz GmbH, Hamburg, Germany) was used. The amplified products were visualized by ethidium bromide staining after gel electrophoresis of 10 *μ*L of the final reaction mixture in 1.5% agarose.

### 2.4. Antimicrobial Susceptibility Testing

Antimicrobial susceptibility tests were performed by the Kirby-Bauer disc diffusion method using Mueller-Hinton agar (HiMedia Laboratories, Mumbai, India, MV1084), according to the Clinical and Laboratory Standards Institute guidelines [41]. After incubating the inoculated plate aerobically at 37°C for 18–24 h in an aerobic atmosphere, the susceptibility of the *E. coli* isolates to each antimicrobial agent was measured, and the results were interpreted in accordance with interpretive criteria provided by CLSI (2006). *E. coli* ATCC 25922 was used as quality control organisms in antimicrobial susceptibility determination.

### 2.5. Statistical Analysis

Statistical analysis were performed using *SPSS/16.0* software for significant relationship between incidences of virulence factors and antibiotics resistance genes of *E*. *coli* isolated from various animal milk samples and dairy products. Statistical significance was regarded at a *P*  value < 0.05.

## 3. Results

In the current study, all *E. coli* colonies were tested by applying PCR method in order to detect 16S rRNA gene of bacterium. According to data, out of 424 animal milk samples, 57 (13.44%) and out of 295 traditional dairy products 45 (15.25%) were positive for presence of *E. coli* (Table 3). Therefore, it was shown that incidences of *E. coli* in different traditional dairy products were higher than animal milk species. Our results showed that bovine (20.9%) and soft cheese (23.58%) had a highest and camel (6.81%) and soft ice cream (10%) had a lowest rate of *E. coli* in raw milk and traditional dairy products, respectively (Table 3). Statistical analysis of data showed significant differences (*P* < 0.05) between bovine, ovine, caprine, and camel samples as well as donkey and camel milk samples for presence of *E. coli*. On the other hand, significant differences (*P* < 0.05) were observed in soft cheese, soft butter, and ice cream in incidence of this bacterium. It was concluded that the total prevalence of *E. coli* in all samples in Iran was 14.18% (102 out of 719).

By applying specific primers for detection of STEC serotypes in various samples, it was indicated that out of 102 positive samples for *E. coli*, 14 (13.72%), 2 (1.96%), 4 (3.92%), 3 (2.94%), 3 (2.94%), 10 (9.8%), 6 (5.88%), 2 (1.96%), 6 (5.88%), and 18 (17.64%) samples were positive for incidences of O157, O145, O128, O121, O113, O111, O103, O91, O45, and O26 serotypes, respectively, while 34 (33.33%) samples have been determined as nondetected serotypes. So the prevalence of O26 was the highest and both O91 and O145 were the lowest of STEC serotypes (Table 3). In addition, O157 (19.29%) and O145 (1.75%) were the highest and lowest serotypes of STEC in raw animal milk samples and O26 (28.88%) and O91 (0.0%) were the highest and lowest serotypes of STEC in traditional dairy products, respectively (Table 3). Statistical analysis of data indicated significant differences (*P* < 0.05) between total presence of O26, O91, 113, 121, 128, and 145 also O157, O91, and 145 serotypes in samples.

Another part of our study was specialized to determine *E. coli's*  virulence genes. Totally *stx1*, *papA*, *cnf1*,* traT*, *cnf2*, and *eae* were the highest and *sfaS* was the lowest virulence genes of *E. coli* which were isolated from raw animal milk samples and traditional dairy products (Table 4). Statistical analysis showed significant differences (*P* < 0.05) between *stx1*, *papA*, and *sfaS* virulence genes which were isolated from raw animal milk samples and traditional dairy products. 

On the other hand, our results revealed that out of 102 *E. coli*-positive samples, 34 (33.33%) were non-STEC and 68 (66.66%) were diagnosed as STEC serotypes (Table 5). Our results showed that raw bovine milk samples and traditional soft cheese had several virulence genes especially *stx1* and *traT*, so were the main sources of pathogenic *E*. *coli* for human in Iran. 

 The last part of our study was allotted to antibiotic resistance patterns in *E. coli* that isolated from raw milk samples and traditional dairy products which evaluated by application of PCR for presences of genes associated with resistance to various antibiotics and disc diffusion method. PCR assays were successfully developed for detection of twelve different resistance genes of genomic DNA of *E. coli* isolates from milk and dairy products. 

Our result indicated that overall the prevalence of resistance to tetracycline (*tetA *+ *tetB*) was 64.70% and both trimethoprim (*dfrA1*) and streptomycin (*aadA1*) were 41.17%, respectively (Table 6). 

 Therefore totally, the prevalence of antimicrobial resistance especially for tetracycline, streptomycin, sulfonamide, chloramphenicol, and finally cephalothin was so high in this survey. 

Besides, statistical analysis revealed a statistical significant association (*P* < 0.05) between *tetA* and *tetB* genes in dairy production, *tetB* with *aac(3)-IV*, *qnr*, *cat1*, and *cmlA* in bovine, ovine, caprine, and buffalo milk samples, *aadA1* with* qnr*, *dfrA1*, *tetB*, *blaSHV*, *CITM*,* cat1*, and *cmlA* in soft cheese, butter, and ice cream. Moreover, our results showed that all STEC serotypes which were isolated from raw animal milk samples and traditional dairy products had resistance to more than one antibiotic. Also, based on our data, out of 102 *E. coli*-positive isolates, all of them were resistant to one or more antimicrobial agent. 

 Finally, antibiogram tests, beside PCR assays, revealed the highest resistance to tetracycline, penicillin, and enrofloxacin with the frequency rate of 58.82%, 46.07%, and 45.09%, respectively, as well as the lowest resistance to nitrofurantoin and ciprofloxacin with the frequency rate of 3.92% and 7.84% (Table 7). On the other hand, in the current situations in Iran, the use of nitrofurantoin and ciprofloxacin due to low antibiotic resistance, can be more effective for treatment of diseases caused by *E. coli*. This survey indicated the highest antimicrobial resistance in O157, O26 and O111 serotypes. Unfortunately, our results showed that nondetected serotypes had a highest incidence of antibiotics resistance. Besides, bovine had highest and camel had lowest rates of resistance to antibiotics. Statistical analysis showed significant differences (*P* < 0.05) between O157, O26, and nondetected serotypes with O103, 145, 91, 113, 121 and 128 serotypes and between resistance to trimethoprim, streptomycin, lincomycin, penicillin, and tetracycline with nitrofurantoin and ciprofloxacin antibiotics. Totally *E. coli* antibiotic resistances against common antibiotics which use in veterinary in Iran were so high. Based on Table 6, between these ten serotypes, *E. coli* O157 and *E. coli* O145 had a highest and lowest prevalence of antibiotic resistance genes. Results showed that in nondetected serotypes, prevalence of antibiotic resistance genes were so high too. 

## 4. Discussion

The present study like the previous studies which introduced ruminants as reservoirs of *E. coli* O157 [42], for the first time, has been introduced donkeys, camels, and buffaloes as the other sources of *E. coli* serotypes especially O157. Despite the highest prevalence of *E. coli* O157 in recent studies [2, 43], our results showed that *E. coli *O26 had a highest frequency rate in traditional dairy products. On the other hand, our study indicated that in spite of raw animal milk samples, pathogenic *E. coli* had a highest prevalence in traditional dairy products. Probably using contaminated water in processing traditional dairy products, infected people who produce dairy products, using contaminated equipments, basic infections of dairy sources which are necessary for producing dairy products, and finally lack of public and individual hygiene, are the factors that can be responsible for the higher prevalence of *E. coli* in traditional dairy products than raw animal milk samples. Only one recent study showed that presence of *E. coli* O157 in foods may be derived from polluted water [44]. 

There are several studies which have detected *E. coli* species in yogurt [45], soft cheeses [46], cheddar cheese [47], and Feta cheese [48]; however in this survey it is for the first time that we observed that this bacterium can be detected in soft cheese, soft butter, and soft ice cream which were produced in traditional conditions too. 

According to Meshref in 2010 from Egypt on cooking butter, 31.7% of the samples were contaminated by *E. coli* [49], in which this rate was higher than ours (11.11%). Another study, performed on dairy products, showed that when the assay was applied with 60 market dairy food samples, one sample each of raw milk, paneer, and ice cream was found to be positive for *E. coli* O157:H7 [50] that was lower than our study. 

 Another study in Iran showed that only *E. coli *O157 subtype was isolated from traditional cheese (4.2%), ice cream (3.33%), and yoghurt (0%) [51] that was more limited than our study. The other study in Pantnagar revealed that out of 135 dairy product samples, 11 samples (8.14%) were positive for *E. coli* [52] that was lower than our study (14.18%). Recent study from Iran indicated that *E. coli *O157:H7 can be detected in high rates from white cheese and probiotic cheese [53]. Besides, a 3-year study of *E. coli* O157:H7 in cattle, camel, sheep, goat, chicken, and beef-minced meat in Iran revealed that 1.7% and 2.1% of samples were positive for *E. coli* O157:H7 and *E. coli *O157:NM, respectively [54]. According to a study in Kerman on raw milk cheese producing, *stx *genes detected in 6.4% of the samples, but STEC strains could be isolated in only five of them (4%) [55]. Another study from Iran showed that about 21.8% of *E. coli *isolates from cattle were positive for *ehxA, stx1*, and/or *stx2 *genes [56], but our study indicated that *stx1*, *cnf1*, and* eae* with incidences of 24.5%, 21.56%, and 17.64% had a highest frequency rate of virulence genes of *E. coli *isolates from raw bovine milk samples.

Therefore, according to the results, the food contamination with animal fecal origin is so obvious in Iran. Based on the other surveys, the presence of *E. coli* O157 in raw milk and dairy products is proven, however in this investigation we found O26, O111, O103, and even O45 in addition to O157 as a predominant serotypes of *E. coli* in animal's raw milk and dairy products with the frequency of 17.64%, 9.8%, 5.88%, 5.88%, and 13.72%, respectively. So in our study *E. coli* O26 was the predominant serotype, AEEC was the predominant subtype, and *papA* and *stx1 *were the predominate genes. 

Another study in Swiss in 2006 on 432 cheese samples and in 2007 on 364 cheese samples showed 16 (3.7%) and 23 (6.3%) *E. coli*-positive samples, respectively, so their results were lower than ours in cheese (23.58%) specimens. Further more in this study the investigators found non-O157 STEC strains in 16 samples in which 11 samples based on their O groups typed into 7 categories (O2, O15, O22, O91, O109, O113, O174), whereas 5 strains were nontypeable. They detect *stx1* in only 1 sample and *stx2* in 15 samples of 16 non-O157 STEC specimens. Furthermore this study showed that 3 STEC strains harbored *E-hlyA* as a further putative virulence factor and none of the strains tested positive for *eae* gene [14]. In another study in Irish bovine dairy herds, 85 out of 117 milk filters and 43 out of 120 bulk-tank milk samples were positive for *vt* genes of *E. coli*. Out of *vt*-positive samples, 58, 162, 1, 324, and 26 samples were positive for O26, O103, O111, O145, and O157 serotypes, and 10 of these isolates contained at least one of the four virulence genes of *vt1*, *vt2*, *eaeA*, and *hlyA* [57]. 

Recent study in United State reported that EHEC subtype of *E. coli* was detected in 23% of strains and *eaeA* gene was found in 4.2%, and one or both Shiga-like toxin genes (*stx1* and *stx2*) were positive in bulk-tank milk too. Based on real-time PCR, they proved that 5 samples were contaminated with O157:H7 [58] while our study showed that out of 102 positive samples for *E. coli*, 13.72% were EHEC, 17.64% had *eaeA *gene, and 35.29% had both Shiga-like toxin genes. 

The prevalence of Shiga toxin in our study (66.66%) was obviously higher than Lowa (4.08%) [59]. Another study on lamb food chain showed that all three virulence genes, *eae*, *stx1*, and *stx2*, were the most prevalent genes in slaughterhouses (69%), processing plant (32%), and butcheries (9-10%) [60], but our results indicated that in raw milk and dairy products, *stx1* and *papA* with frequency rate of 24.5% had a highest prevalence of *E. coli*'s virulence genes.

Detection of bacteria in milk samples has some merits like being available and economical. In some geographic places of Iran because of bad climate conditions, bovines, ovine, caprine, and buffaloes are not able to survive, so camels are the main milk supplier for people. So camel's milk hygiene can be essential for human health especially in those especially in that area where the climate is arid and desert. Donkeys' milk samples due to high similarity with human milk and high-sweet components due to high presence of lactose sugar sometimes can be used instead of human milk. Buffaloes' milk hygiene due to high fat and protein contents is very important especially for producing some dairy products. 

Studies showed that the antibiotic resistance bacteria which exist in the milk of infected animals can be transmitted to human by the ingestion of unpasteurized milk or dairy products such as cheese, butter, yoghurt, and ice cream. However, the mechanism of spreading antibiotic resistance from food animals to humans remains controversial [61, 62]. 

Our study showed that *E. coli* O157 was the most prevalent serotype in donkey's milk. Furthermore, this serotype had the highest antibiotic resistance to penicillin and enrofloxacin. To our knowledge, veterinary service-like treatments are very low for donkeys in Iran and unfortunately if a veterinarian wants to apply antibiotical treatments, in most of cases uses penicillin and enrofloxacin for donkeys. Therefore, the high antibiotic resistance in donkeys to these two antibiotics (in our study) may be occurred due to these successive and ectopic treatments, but in bovines the best choices for antibiotic treatment were enrofloxacin, trimethoprim, and tetracycline. So due to the high administration of antibiotics, the prevalence of antibiotic resistance against these antibiotics was so high in bovine's milk samples in the current study. Veterinary interventions and antibiotic administrations are not common in camels in Iran. In most regions of Iran, camel's distribution is so low and reports about the occurrences of diseases in camels usually were low in Iran. Therefore based on our results, it seems logical that the prevalence of antibiotics resistance in camel samples was low. 

Our results showed that 58.82% of isolated *E. coli* from raw milk and traditional dairy products were tetracycline resistant. As well as an other study, substantial numbers of human *E. coli* isolates were tetracycline resistant [63]. Recent study indicated that 61% of *E. coli* which isolated from humans, cattle, swine, and food during 1985 to 2000 was susceptible to all 13 antimicrobials tested, while 27% of them were resistant to tetracycline, 26% to sulfamethoxazole, 17% to cephalothin, and 13% to ampicillin [63]. As the present study indicates, there is a high antibiotic resistance in *E. coli* which isolated from raw milk and traditional dairy products. 

A report from Scotland revealed that the frequency of antibiotic-resistant *E. coli* was 10.6% in milk samples and suggested that the prevalence of antibiotic-resistant strains in milk was higher in housed bovines than in outdoor bovines. This study showed that out of 1125 *E. coli* isolates tested, 22.2% were antibiotic resistant, and out of these resistant strains, 42.4% were resistant to more than one antibiotic [64]. Another study in Iran indicated that the resistance of the *E. coli *strains isolated from traditional cheese, ice cream, and yoghurt to ampicillin and gentamycin was the most common findings (44.4%), followed by resistance to erythromycin (33.3%), amoxicillin (11.1%), tetracycline (11.1%), and nalidixic acid (11.1%) [51]. Our study showed that two of the most prevalent resistant genes of *E. coli* were *tetA *+ *tetB* (64.70%), and this is similar to previous study that was performed on cow's milk [65]. 

Unfortunately, indiscriminate prescribing of antibiotics in veterinary is so high and in a report from Netherland 300 000 kg of antibiotics are used annually on veterinary prescription in animals [66]. These high amounts of antibiotics can be kept in animals and lead to antibiotics resistance in bacteria. Then resistance genes of bacteria shedding from milk and meat can be used in humans. So these resistance genes will be causing drug resistance in human too. Our results showed that simultaneous application of culture and PCR can easily be effective in diagnosis of pathogens and their resistance factors especially in food for human consumption. 

Our results revealed that (i) using raw milk without pasteurization and milking with unsanitary methods and using traditional dairy products that are produced in unsanitary conditions and probably from unpasteurized milk are the main resources for growth, proliferation, and survival of *E. coli* and cause several disorders for human. Therefore, vaccinating dairy animals (if necessary), improving methods of milking, checking milking halls in order to detect *E. coli* especially in the animal feces monthly, fumigating milking halls frequently, observing hygiene during milking, boiling milk, using pasteurized milk for dairy products, keeping dairy products in cool and dry places, away from sunlight, and finally preventing contamination of dairy products with extrinsic factors like insects and dust are the best ways to prevent *E. coli* infections. (ii) Chloramphenicol is a forbidden antibiotic, and the high antibiotic resistance to drug in our study indicated the irregular and unauthorized use of this antibiotic, in veterinary treatment in Iran. Unfortunately, veterinarians in many fields of veterinary such as large animal internal medicine, poultry, and even aquaculture use this antibiotic as a basic one. Therefore, in a very short period of time, antibiotic resistance will appear. (iii) We recommend PCR method as an accurate, safe, and fast diagnostic one for detection of pathogens in milk and dairy products (iv) due to antibiotic resistance especially in *E. coli*, the veterinarians should pay more attention to prescribing the antibiotics. (v) *E. coli* virulence genes especially *stx1*, *stx2*, *eae*, *cnf1*, *cnf2*, *iutA*, *papA*, and finally *traT* are well distributed in milk and dairy products in Iran. (vi) STEC serotypes and especially AEEC are the predominant strains and O157, and O26 serotypes are the predominant serotypes of bacterium in animal raw milk and traditional dairy products in Iran. (vii) Bovine and camel raw milk and soft cheese and ice cream had a highest and lowest frequency rate of pathogenic *E. coli* in raw milk and dairy product samples in Iran, respectively. (viii) In order to prevent antibiotic resistance in bacteria, we should apply antibiotics more cautiously in animals, detect resistance genes, and finally use different antibiotics periodically. Our results recommended the use of PCR for detection of antibiotic resistance genes of bacteria as a safe, rapid, and accurate method in laboratories.

## Figures and Tables

**Table 1:** Primer sequence for detection of STEC serotypes in animals milk and different traditional dairy products.

Primer name	Sequence	Size of product (bp)	Target gene	Reference
O26-F O26-R	CAG AAT GGT TAT GCT ACT GT CTT ACA TTT GTT TTC GGC ATC	423	*wzx*	[25]
O103-F O103-R	TTGGAGCGTTAACTGGACCT GCTCCCGAGCACGTATAAG	321	*wzx*	[25]
O111-F O111-R	TAG AGA AAT TAT CAA GTT AGT TCC ATA GTT ATG AAC ATC TTG TTT AGC	406	*wzx*	[25]
O145-F O145-R	CCATCAACAGATTTAGGAGTG TTTCTACCGCGAATCTATC	609	*wzx*	[25]
O157-F O157-R	CGG ACA TCC ATG TGA TAT GG TTG CCT ATG TAC AGC TAA TCC	259	*wzx*	[25]
O45-F O45-R	CCG GGT TTC GAT TTG TGA AGG TTG CAC AAC AGC CAC TAC TAG GCA GAA	527	*wzx1*	[26]
O91-F O91-R	GCTGACCTTCATGATCTGTTGA TAATTTAACCCGTAGAATCGCTGC	291	*gnd*	[27]
O113-F O113-R	GGGTTAGATGGAGCGCTATTGAGA AGGTCACCCTCTGAATTATGGCAG	771	*wzx*	[19]
O121-F O121-R	TGGCTAGTGGCATTCTGATG TGATACTTTAGCCGCCCTTG	322	*wzx*	[28]
O128-F O128-R	GCTTTCTGCCGATATTTGGC CCGACGGACTGATGCCGGTGATT	289	*galF*	[29]

**Table 2:** *E. coli*-resistant antibiotic genes, primer sequence, and product size.

Antibiotic	Resistant gene	Sequence	Size (bp)	Annealing temperature (°C)	References
Streptomycin	*aadA1*	(F) TATCCAGCTAAGCGCGAACT (R) ATTTGCCGACTACCTTGGTC	447	58	[30]
Tetracycline	*tetA*	(F) GGTTCACTCGAACGACGTCA (R) CTGTCCGACAAGTTGCATGA	577	57	[30]
Tetracycline	*tetB*	(F) CCTCAGCTTCTCAACGCGTG (R) GCACCTTGCTGATGACTCTT	634	56	[30]
Trimethoprim	*dfrA1*	(F) GGAGTGCCAAAGGTGAACAGC (R) GAGGCGAAGTCTTGGGTAAAAAC	367	45	[31]
Fluoroquinolone	*qnr*	(F) GGGTATGGATATTATTGATAAAG (R) CTAATCCGGCAGCACTATTTA	670	50	[32]
Gentamicin	*aac(3)-IV*	(F) CTTCAGGATGGCAAGTTGGT (R) TCATCTCGTTCTCCGCTCAT	286	55	[33]
Sulfonamide	*Sul1*	(F) TTCGGCATTCTGAATCTCAC (R) ATGATCTAACCCTCGGTCTC	822	47	[33]
Cephalothin	*blaSHV*	(F) TCGCCTGTGTATTATCTCCC (R) CGCAGATAAATCACCACAATG	768	52	[33]
Ampicillin	*CITM*	(F) TGGCCAGAACTGACAGGCAAA (R) TTTCTCCTGAACGTGGCTGGC	462	47	[33]
Chloramphenicol	*cat1*	(F) AGTTGCTCAATGTACCTATAACC (R) TTGTAATTCATTAAGCATTCTGCC	547	55	[33]
Chloramphenicol	*cmlA*	(F) CCGCCACGGTGTTGTTGTTATC (R) CACCTTGCCTGCCCATCATTAG	698	55	[33]

**Table 3:** Prevalence of STEC serotypes isolated from raw animal milk samples and traditional dairy products in Iran.

Samples	*E. coli* positive (%)	O157 (%)	O145 (%)	O128 (%)	O121 (%)	O113 (%)	O111 (%)	O103 (%)	O91 (%)	O45 (%)	O26 (%)	Not detected (%)
Bovine milk (110)	23 (20.9)	3 (13.04)	1 (4.34)	2 (8.69)	1 (4.34)	1 (4.34)	3 (13.04)	1 (4.34)	1 (4.34)	3 (13.04)	2 (8.69)	5 (21.73)
Ovine milk (98)	9 (9.18)	1 (11.11)	—	—	—	—	—	1 (11.11)	1 (11.11)	1 (11.11)	1 (11.11)	4 (44.44)
Caprine milk (85)	8 (9.41)	1 (12.5)	—	1 (12.5)	—	—	1 (12.5)	—	—	1 (12.5)	1 (12.5)	3 (37.5)
Buffalo milk (52)	8 (15.38)	1 (12.5)	—	—	1 (12.5)	—	1 (12.5)	—	—	—	1 (12.5)	4 (50)
Camel milk (44)	3 (6.81)	1 (33.33)	—	—	—	1 (33.33)	—	—	—	—	—	1 (33.33)
Donkey milk (35)	6 (17.14)	4 (66.66)	—	—	—	—	1 (16.66)	—	—	—	—	1 (16.66)
Total milk (424)	57 (13.44)	11 (19.29)	1 (1.75)	3 (5.26)	2 (3.5)	2 (3.5)	6 (10.52)	2 (3.5)	2 (3.5)	5 (8.77)	5 (8.77)	18 (31.57)
Soft cheese (106)	25 (23.58)	2 (8)	1 (4)	1 (4)	1 (4)	1 (4)	2 (8)	1 (4)	—	1 (4)	8 (32)	7 (28)
Soft butter (99)	11 (11.11)	1 (9.09)	—	—	—	—	1 (9.09)	1 (9.09)	—	—	3 (27.27)	5 (45.45)
Soft ice cream (90)	9 (10)	—	—	—	—	—	1 (11.11)	2 (22.22)	—	—	2 (22.22)	4 (44.44)
Total dairy products (295)	45 (15.25)	3 (6.66)	1 (2.22)	1 (2.22)	1 (2.22)	1 (2.22)	4 (8.88)	4 (8.88)	—	1 (2.22)	13 (28.88)	16 (35.55)

Total (719)	102 (14.18)	14 (13.72)	2 (1.96)	4 (3.92)	3 (2.94)	3 (2.94)	10 (9.8)	6 (5.88)	2 (1.96)	6 (5.88)	18 (17.64)	34 (33.33)

**Table 4:** Distribution of virulence genes in *E. coli* isolated from raw animal milk samples and traditional dairy products in Iran.

Samples	Virulence genes											
	*stx1*	*stx2*	*eae*	*ehly*	*cnf1*	*cnf2*	*iutA*	*cdtB*	*papA*	*traT*	*sfaS*	*fyuA*
Bovine milk	9	3	4	1	4	3	2	2	2	3	—	1
Ovine milk	3	1	2	—	2	1	5	—	2	2	—	—
Caprine milk	3	1	1	—	2	1	1	—	5	2	—	—
Buffalo milk	4	2	3	1	2	4	2	1	2	2	—	—
Camel milk	2	1	1	2	1	3	—	2	5	1	—	1
Donkey milk	1	1	1	2	6	1	—	1	1	3	1	2
Total milk	22	9	12	6	17	13	10	6	17	13	1	4
Soft cheese	2	1	2	2	2	1	2	—	2	6	—	—
Soft butter	—	—	2	1	2	1	2	1	2	1	—	—
Soft ice cream	1	1	2	—	1	3	1	1	4	1	—	—
Total dairy products	3	2	6	3	5	5	5	2	8	8	—	—

Total	25	11	18	9	22	18	15	8	25	21	1	4

**Table 5:** Prevalence of virulence genes in Shiga toxin-producing *E. coli* serotypes isolated from raw animal milk samples and traditional dairy products in Iran.

Serotype		Number of positive samples (%)	Virulence gene (%)
Non-STEC		34 (33.33)	—
STEC (68 = 66.66%)	EHEC	14 (20.58)	*stx1*, *eae*, *ehly*: 14 (100)
	AEEC	54 (79.41)	*stx1*: 25 (46.29) *stx2*: 11 (20.37) *eae*: 18 (33.33) *stx1*, *eae*: 12 (22.22) *stx2*, *eae*: 7 (12.96) *stx1*, *stx2*, *eae*: 4 (7.4)

**Table 6:** Distribution of antibiotic resistance genes in various serotypes of *E. coli* isolated from raw animal milk samples and traditional dairy products in Iran.

STEC serotypes	Antibiotic resistance genes										
	*aadA1*	*tetA*	*tetB*	*dfrA1*	*qnr*	*aac(3)-IV*	*sul1*	*blaSHV*	*CITM*	*cat1*	*cmlA*
O157 (14)	10	7	5	12	14	7	8	7	8	6	4
O26 (18)	13	11	4	5	5	8	11	10	9	3	8
O103 (6)	1	2	1	2	2	1	2	2	2	1	—
O111 (10)	3	3	2	7	12	1	4	6	4	4	1
O145 (2)	—	—	—	—	—	—	1	—	—	—	1
O45 (6)	1	1	—	6	1	—	1	3	5	1	—
O91 (2)	—	—	1	1	—	1	1	—	—	—	—
O113 (3)	—	1	—	1	—	1	1	1	1	—	1
O121 (3)	—	—	1	—	—	—	1	—	1	1	—
O128 (4)	1	2	—	1	—	—	1	1	1	1	1
Not detected (34)	13	10	15	7	26	9	12	13	9	6	8

Total (102)	42 (41.17)	37 (36.27)	29 (28.43)	42 (41.17)	60 (58.82)	28 (27.45)	43 (42.15)	43 (42.15)	40 (39.21)	23 (22.54)	24 (23.52)

**Table 7:** Antibiotic resistance properties in STEC serotypes isolated from raw animal milk and traditional dairy products in Iran (disk diffusion method).

STES serotypes	P10 (%)	TE30 (%)	S10 (%)	C30 (%)	SXT (%)	GM10 (%)	NFX5 (%)	L2 (%)	CF30 (%)	CIP5 (%)	TMP5 (%)	F/M300 (%)	AM10 (%)
O157 (14)	12	11	9	9	8	5	12	9	6	1	10	1	6
O26 (18)	4	14	12	9	10	6	3	10	8	1	5	1	8
O103 (6)	—	3	1	—	1	—	1	2	2	—	2	—	2
O111 (10)	8	4	3	4	3	1	9	3	5	2	7	—	3
O145 (2)	—	—	—	—	1	—	—	—	—	—	—	—	—
O45 (6)	4	1	2	1	1	—	1	1	2	—	5	—	1
O91 (2)	—	—	—	—	—	—	—	—	—	—	1	—	—
O113 (3)	—	1	—	—	—	1	—	—	—	—	1	—	1
O121 (3)	1	1	—	—	1	—	—	1	—	—	—	—	1
O128 (4)	1	1	1	1	—	—	—	1	1	—	1	—	1
Not detected (34)	17	24	10	12	7	7	20	15	11	4	9	2	4

Total (102)	47 (46.07)	60 (58.82)	38 (37.25)	36 (35.29)	32 (31.37)	20 (19.6)	46 (45.09)	42 (41.17)	35 (34.31)	8 (7.84)	41 (40.19)	4 (3.92)	27 (26.47)

In this table P10: penicillin (10 u/disk); TE30: tetracycline (30 *μ*g/disk); S10: streptomycin (10 *μ*g/disk); C30: chloramphenicol (30 *μ*g/disk); SXT: sulfamethoxazole (25 *μ*g/disk); GM10: gentamycin (10 *μ*g/disk); NFX5: enrofloxacin (5 *μ*g/disk); L2: lincomycin (2 *μ*g/disk); CF30: cephalothin (30 *μ*g/disk); CIP5: ciprofloxacin (5 *μ*g/disk); TMP5: trimethoprim (5 *μ*g/disk); F/M300: nitrofurantoin (300 *μ*g/disk); AM10: ampicillin (10 u/disk).
